# NM GRADS: Lessons Learned from Implementing a School-Based Program for Young Parents Across New Mexico

**DOI:** 10.1007/s10995-020-02993-5

**Published:** 2020-08-29

**Authors:** Jessica Harper, Dean Hopper, Betsy Keating, Jessica Harding

**Affiliations:** 1 New Mexico Public Education Department, 120 South Federal Place, Room 206, Santa Fe, NM 87501 USA; 2 Mathematica, P.O. Box 2393, Princeton, NJ 08543 USA

**Keywords:** School-based supports, Young parents, Expectant and parenting students, Graduation rates, Repeat birth, Repeat pregnancy, Teen parents

## Abstract

**Purpose:**

The New Mexico Graduation Reality and Dual-role Skills (GRADS) program provides services for expectant and parenting students at high schools. The GRADS program has operated since 1989, serving more than 17,000 youth. This study summarizes the GRADS program model and program administrators’ lessons learned from implementing this comprehensive, large-scale program.

**Description:**

The GRADS program is a multicomponent intervention that can include a classroom intervention, case management, linkages to child care and health care, and support for young fathers. The program aims to support expectant and parenting youth in finishing high school, delaying a repeat pregnancy, promoting health outcomes for their children, and preparing for college and career. This study presents program administrators’ lessons learned to increase understanding of how to implement a statewide program to support expectant and parenting students.

**Assessment:**

During the 2010–2017 school years, the GRADS program operated in 26–31 sites each year, serving a total of 2691 parenting youth. Program administrators identified lessons learned from implementing the GRADS program during that period of expansion, including allowing variation across sites based on resources and needs, providing centralized implementation support, fostering buy-in from school and district leaders, and collecting consistent data to better understand participant outcomes.

**Conclusions:**

Although not based on a rigorous impact or implementation study, this article provides lessons learned from a statewide, school-based program that may be a promising way to serve a large number of expectant and parenting youth and help them overcome challenges for completing high school.

## Significance

*What is already known on this subject*? Some school-based programs implemented across a small set of schools have been found to be effective in promoting outcomes for expectant and parenting youth.

*What this study adds*? This article provides lessons learned from statewide implementation of a potentially promising school-based program to support expectant and parenting youth. Although this paper does not assess the effectiveness of the program, it illuminates important information for researchers and practitioners about a program that is flexible in addressing the range of multifaceted needs of expectant and parenting youth.

## Purpose

Although the national teen birth rate has decreased significantly over the past several decades, New Mexico continues to have a high rate, with 27.9 births per 1000 females ages 15–19, compared to 18.8 for the United States as a whole (Centers for Disease Control and Prevention 2018). Teen births are particularly pronounced in some areas of the state: eight New Mexico counties had teen birth rates of more than 40 births per 1000 females ages 15–19 (New Mexico Department of Health 2018).

In balancing their responsibilities as students and parents, expectant and parenting youth (EPY) face myriad challenges to high school graduation. Barriers may include lack of child care, financial struggles, housing instability, and limited access to health care (Annie E. Casey Foundation 2018). Perhaps because of these challenges, EPY are less likely than their peers to graduate from high school and attend college (National Conference of State Legislators 2013; Lee 2010; Hofferth et al. 2001; Fletcher and Wolfe 2009, 2012). In a survey of youth who dropped out of high school, 26% of those surveyed reported that becoming a parent was a primary factor in their decision to leave school (Bridgeland et al. 2006). EPY who experience a repeat pregnancy during their teenage years are even less likely to complete high school (Klerman 2004; Schuyler Center for Analysis and Advocacy 2008).

School-based programs can support young parents in completing high school and delaying a repeat pregnancy. Such programs often provide a coordinated system of support for EPY, building their academic skills while also offering additional support services. For instance, some school-based programs provide high-quality child care, which helps eliminate one key barrier to teen parents’ school attendance (Sadler et al. 2007; Van Pelt 2012). Research suggests school-based programs improve teens’ academic outcomes (Amin and Sato 2004; Amin et al. 2006; Barnet et al. 2004; Crean et al. 2001; Harris and Franklin 2003). For example, a recent study in this journal supplement found that a school-based program for young parents had positive impacts on school engagement and graduation rates (Zief et al. 2020 in this issue). However, some of these programs include only a single component, such as student health centers, to support teen parents. Limited research has described the components of multifaceted school-based programs that are flexible to address the complex needs of EPY.

Since 1989, the New Mexico Graduation Reality and Dual-role Skills (GRADS) program has provided school-based services to address the multifaceted needs of more than 17,000 EPY (NM GRADS 2017). The primary purpose of the program is to increase high school graduation rates among EPY, and a secondary goal is to delay repeat pregnancies. Other program goals include encouraging prenatal care to promote healthy birth weight; improving child development outcomes such as school readiness; helping participants balance family, school, and work roles; preparing participants for careers and economic independence; and promoting healthy family relationships. With funding from the U.S. Department of Health and Human Services, Pregnancy Assistance Fund (PAF) from 2010 to 2017, the New Mexico Public Education Department (PED) expanded the services offered in the GRADS program and the number of schools implementing GRADS. During the PAF funding period, the GRADS program was widely implemented across the state, with between 26 to 31 sites each year. A total of 36 sites implemented GRADS across all PAF funding years, from 27 of the state’s 89 school districts (30% of districts).

This paper describes the approach of the GRADS program and program administrators’ lessons learned from implementation during the 2010–2017 PAF funding and expansion period. Because some smaller scale school-based programs have been found to be effective in promoting EPY outcomes (Asheer et al. 2017), this paper can provide important information for researchers and practitioners about a statewide implementation of a potentially promising school-based program to support EPY. In general, researchers do not fully understand the program components that support a successful program for EPY in school. Although this paper does not attempt to demonstrate the impact of the program, it does describe a potentially promising program that is flexible in addressing the range of multifaceted needs of EPY and the different schools they attend. In particular, this article highlights key lessons learned from program administrators about implementing a multicomponent intervention that aims to meet the unique needs of EPY.

## Description

The GRADS program provides multifaceted support for EPY in high school. A state-level GRADS office oversees program implementation across the sites and serves as the primary point of contact for them. Program sites are typically high schools, although one site is a community-based organization. All sites serve youth enrolled in school, and some sites continue to offer services like case management after youth have graduated from high school. As we discuss in more depth in the following sections, the program includes a classroom intervention, case management, and additional services intended to ease many of the barriers that EPY face in graduating from high school. The availability of some services varies by site based on participants’ needs and the site’s available resources; these services may include child care, access to health care, and academic support. Young parents may be referred to additional services such as home visiting, early intervention services, behavioral health services, workforce training, and housing.

The GRADS program operates as a partnership between PED, the state-level GRADS office, and local implementation sites. The PED plans and oversees program funding and sustainability; provides ongoing programmatic consultation and guidance; and manages several statewide contractors, including Fathers New Mexico and the New Mexico Alliance for School-Based Health Care, which provide technical assistance and support implementation. The state GRADS office manages the program across the sites while district and school staff facilitate program implementation at each site. The state GRADS office, with input from PED, supports program staff through three in-person trainings per school year, giving guidance on implementing the GRADS classroom intervention, case management services, and onsite child care and providing technical assistance throughout the year to troubleshoot issues. Staff from the state GRADS office visit each program site at least once every school year to provide in-person assistance and support; new sites receive at least two visits during their 1st year of implementation. PED and the state GRADS office work together to develop data collection tools, analyze data, and determine whether any changes to the program are needed. At each site, district and school leaders approve the program and hire all staff working on the GRADS program.

Staff composition varies at each GRADS site depending on the services offered, but all sites have a GRADS teacher who provides the classroom intervention or other program components. The classroom teacher is often a family and consumer sciences teacher, devoting at least one period of his or her day to the GRADS program. Sites may also hire case managers and fatherhood mentors to provide services to students. With increased funding through the PAF grant, program administrators reported more sites were able to hire additional staff and offer more intensive services.

### Components of the GRADS Intervention

In this section, we describe the different components of GRADS, some of which vary by site according to site needs and resources. During the PAF funding period, the PED and state GRADS office expected all program sites to implement the following program components: the classroom intervention, comprehensive case management, linkages to health care through school-based health centers (SBHCs) or in the community, linkages to child care, and support for young fathers.^1^ The intensity and exact implementation of each component might vary by site—for instance, some sites offered onsite child care at the school while others referred students to child care services in the community. Table 1 indicates the number of sites that had onsite case managers, child care centers, and SBHCs. Through a coordinated approach, the program components can work together to help students reach goals in education, economic independence, family planning and health (including delaying second pregnancies), parenting, and safe and healthy relationships.

**Table 1:** Availability of onsite case manager, child care center, and SBHC at the 36 sites during the PAF funding period in 2010–2017^a^

Onsite components	Number of GRADS sites
No onsite case manager, child care center, or SBHC	2
*One onsite component*	
Onsite case manager only	4^b^
Onsite child care center only	3
Onsite SBHC only	2
*Two onsite components*	
Onsite case manager and SBHC only	4^b^
Onsite case manager and child care center only	5
Onsite child care center and SBHC only	6
*Three onsite components*	
Onsite case manager, child care center, and SBHC	10^c^
Total GRADS sites	36

^a^During the PAF funding period, the program had between 26 and 31 sites each year; these sites varied slightly across the funding years. In all, there were 36 sites that operated GRADS at some point between the 2010–2011 and 2016–2017 school years. This table summarizes the components implemented onsite at each of those 36 sites

^b^One site did not offer the classroom intervention

^c^At one site, students had access to an SBHC at a nearby school but not at in their school building. A second site did not offer the classroom intervention

### Classroom Intervention

With a focus on college and career readiness, the GRADS classroom intervention is a primary component of the GRADS program. Students can take the class for up to four years as an elective course and receive credit for participation. GRADS classroom intervention implementation schedules vary by site, with some sites having a 50-min class every day and others having a 90-min class a few times a week, but all must meet the state minimum requirements for class length. Participants receive a variety of resources and support to stay in high school, graduate, pursue postsecondary education and career opportunities, and attain self-sufficiency. Specifically, the GRADS class focuses on 10 competencies, including personal development, pregnancy and wellness, postpartum and neonatal care, parenting, child development, relationships, economic independence, and career development. Each GRADS student has a notebook that includes activities and content in each of the 10 competencies.

To address the 10 competencies, the intervention has specific content for teachers to follow. For instance, the intervention includes *FLASH*, a comprehensive sexual education curriculum that covers topics such as pregnancy prevention, sexually transmitted infections, healthy decision making and communication skills, abstinence, and birth control (FLASH 2019). With *FLASH*, the classroom intervention aims to address the program’s goals around delaying second pregnancies and promoting healthy relationships. Additionally, the GRADS intervention includes a career readiness curriculum and resources to help teen parents build skills and prepare for their careers. GRADS students develop career portfolios and complete career assessments and inventories to develop educational goals and post-graduation plans. To connect EPY to services and opportunities for career or educational advancement in their community, program staff develop partnerships with school counselors, workforce partners, career technical education programs, and institutions of higher education. The program also encourages the classroom teacher to implement other curricula, such as the *Adolescent Parent Resource Guide*, *Dibble Love Notes*, *Dibble Money Habitude*, *24/7 Dads*, *Soft Skills in the Workplace*, and *Circle of Security Parenting*.

### Access to School-Based Health Centers or Community Health Care

The GRADS staff support youth in receiving health care, either at SBHCs or other community organizations. During the PAF funding period, 23 GRADS sites had SBHCs, which provide onsite, integrated medical, reproductive, behavioral, and preventive health services.^2^ The exact services provided by SBHCs vary by site based on their available resources. GRADS staff also provide referrals to local health providers for prenatal care, family planning, primary care services, and well-child care, particularly in sites without SBHCs. This program component intends to encourage prenatal and maternal health care, and health outcomes for the participants’ children. To build capacity for this program component during the PAF funding period, PED contracted with the New Mexico Alliance for School-Based Health Care to provide technical assistance to GRADS sites on improving young parent health literacy, school health care coordination, and use of SBHCs and other community health services.

### Comprehensive Case Management

Comprehensive case management links students with services and supports. During the PAF funding period, 23 GRADS sites had onsite case managers that provided one-on-one support to EPY. In sites that could not hire a case manager, the classroom teacher conducted case management. The frequency and length of case management sessions vary by site, based on staff availability, the number of youth in the program, and specific student needs.

The GRADS case management model has a formalized process for service coordination among students, families, teachers, SBHCs, school nurses, and social workers. Case managers use an assessment tool to identify young parents’ needs in the following areas: basic needs, education, college/career readiness, employment assistance, physical health, emotional/behavioral health, reproductive health, child care needs, and legal assistance. Youth are engaged in the needs assessment process so that they have an active role in identifying their own needs and timelines for addressing those needs. From the assessment, the case manager can identify high-priority needs for each young parent and then refer youth to additional services or support to meet those needs. The referrals can include government services such as Medicaid, Temporary Assistance for Needy Families, Special Supplemental Nutrition Program for Women, Infants, and Children, and child support; health services for young parents and well-child care for their children through the SBHC or other community health resources (as previously discussed); behavioral health services; concrete supports for basic needs such as food, clothing, transportation and housing; early childhood services (discussed more below); academic support and planning; and workforce training and supports.

In addition, case managers focus on improving participants’ school outcomes by monitoring attendance and academic performance and by assisting youth in addressing challenges with attending school or completing schoolwork. They also support participants in making up missed material from school absences related to medical appointments, childbirth, or extended hospital stays.

### Onsite Child Care and Linkages to Community Child Care

Recognizing that a lack of child care can be a substantial barrier to completing high school for young parents, the GRADS program supports participants in finding child care services. At 24 GRADS sites, licensed Child Development Centers provided onsite child care for GRADS program participants during the PAF funding period. In sites without onsite child care centers, GRADS staff linked young parents to community child care centers. Through child care subsidies administered by the New Mexico Children, Youth & Families Department, GRADS participants can be reimbursed for their child care expenses. At all sites, GRADS staff can also provide referrals to participants to early childhood services like home visiting and early intervention services, based on participant needs.

In some sites with onsite child care, GRADS participants can work part-time at the child care center and earn child development credit hours. These positions give EPY the opportunity to observe child care center staff modeling effective parenting and to learn successful child care strategies. Working in the child care centers also allows EPY to apply the parenting skills and child development information taught in the classroom intervention.

### Onsite Services for Young Fathers

All program sites are expected to provide tailored support for young fathers, but the intensity of these services may vary by site. To serve young fathers effectively, more than half of the GRADS sites hired a fatherhood mentor to provide onsite fatherhood services during the PAF funding period. Mentors provide young fathers with ongoing support related to staying in school and preparing for graduation to address the particular challenges that young fathers may face. Services include outreach to fathers, individual case management sessions, and fatherhood groups. Fatherhood groups meet once or twice a month and may cover topics like communication and parenting skills, negotiating relationships with the mother of the child, and setting academic goals. This program component is supported by statewide contractors, such as Fathers New Mexico, who provide technical assistance and other support to GRADS sites.

## Assessment

Although GRADS is a longstanding program, these lessons learned focus on the 2010–2017 PAF funding period. During each year of the PAF funding period, the program had between 26 and 31 sites each year (36 total sites), including in some of the largest metropolitan areas like Albuquerque and Las Cruces. Most program sites had a GRADS program prior to 2010 and may have enhanced existing components during the PAF funding period. Eleven sites were onboarded during the PAF funding period.^3^ According to GRADS program records, the program served 2691 unique youth across program sites during the PAF funding period; youth can participate in the program for multiple years throughout high school and can receive ongoing case management services until age 24.^4^ In addition, the onsite child care centers at schools served 1326 children of the GRADS program participants over the same time period. The large-scale implementation of the GRADS program across the 36 sites during the PAF funding period provides information about key lessons from program administrators about their implementation experiences that can inform other organizations considering implementing a school-based program to support teen parents.

Lessons learned from program administrators during the implementation of the GRADS program can inform other practitioners or policymakers interested in serving EPY through a multicomponent, school-based program.

### Lesson One: When Expanding to Serve a Diverse Set of Communities, Allow for Variation Across Sites, Depending on Site Needs and Resources

PED and the state GRADS office allow for variation in the specific components implemented at each GRADS site, and sites can decide how to adopt them based on their resources and participant needs. For instance, if site staff know they have a large number of young fathers that need additional support, they can have a more robust fatherhood services component, including hiring a fatherhood mentor. Similarly, for the case management services, sites can determine whether to hire a separate case manager or if the GRADS classroom teacher will serve as the case manager in addition to providing the classroom intervention. One program administrator explained that this flexibility allowed sites to implement the GRADS program even if they were not able to hire additional support staff like case managers.

With the flexibility of GRADS program delivery, sites can tailor the program to their population and even choose to augment the GRADS program with more services. In one site, school staff recognized participants had a particular challenge with transportation to school, so they added transportation services for GRADS participants. Likewise, sites can tailor services to fit the particular cultural needs of their youth. For example, one site that predominantly serves Navajo youth provides culturally responsive onsite child care by displaying the names of colors and numbers in the Navajo language (Diné Bizaad) and including Navajo cultural objects in the child care center. Other sites offer Spanish-language materials or support, if needed.

### Lesson Two: Provide Centralized Support for Program Implementation

Although GRADS is a relatively diffuse program with sites across the state of New Mexico, the PED and state GRADS office staff help to provide centralized support for all GRADS sites. PED and the state GRADS office have institutional knowledge of the program that they share with sites at the start of the program or as sites face new implementation challenges. Prior to each school year, the state GRADS office trains any new teachers and staff on the program, offering a variety of resources and curricula to support implementation. Following that, the GRADS classroom teachers or other site staff can reach out to the state office any time they have questions or concerns, which can be particularly helpful for staff new to the program. All GRADS teachers participate in annual training, receive ongoing technical assistance, and can access a variety of resources to assist them in addressing the 10 competencies and meeting student learning goals. In addition, the state office harnesses the experience and expertise of the longtime GRADS teachers to support implementation. For instance, veteran GRADS classroom teachers serve as regional mentors and share expertise with newer staff. As described earlier, the state GRADS office and PED have also supported additional consultants and TA providers to provide support to teachers at various times.

The state GRADS office has a technical assistance coordinator, who visits all sites at least once, with new sites receiving multiple visits in their 1st year of implementation. Each visit involves an onsite review of the program with the classroom teacher and other staff as available, to ensure effective program implementation and improve the program over time. During these visits, state office staff discuss strengths and areas of improvement with local GRADS staff in order to meet the needs of EPY in the following areas: implementation of GRADS program components, data submission, participating in trainings and technical assistance opportunities, and collaboration with school and community partners at that site. The site visit may also include a review of the GRADS students’ notebooks to ensure that the classroom intervention is addressing the 10 competencies. GRADS state staff may have conversations with the principal about program effectiveness, including whether the teacher is the best fit for the program. As one program administrator noted, this on-the-ground support allows state staff to better understand the resources and needs of each site and to provide tailored recommendations to each site.

### Lesson Three: Build and Maintain Local Buy-in to the Program to Support Sustainability

The state GRADS office dedicates time to build and sustain buy-in from school and district leaders for the GRADS program. When first recruiting schools, the state office highlights the GRADS program’s goal of helping young parents graduate, which mirrors the principal’s or superintendent’s own goals around supporting the academic success of all students, including young parents. Understanding their shared goals helps school leaders become more invested in the program, strengthening the program’s sustainability efforts. In addition, program administrators noted that having a planning period to onboard sites was helpful. During the planning period, they invite school and district leaders to events and trainings, so they fully understand the program before it comes to their school. As part of the planning period, the state GRADS office asks school and district leaders to select the GRADS classroom teacher and other staff. By working to identify staff that will work best for the GRADS program, school and district leaders gain greater ownership in the program and better understand its role in their schools. With strong support and buy-in for the program, principals and superintendents can provide on-the-ground leadership.

Likewise, the state office and PED continue to engage with these local leaders to maintain their support for GRADS over time. This strategy is particularly important for addressing the challenge of turnover among school or district leaders. Because new administrators may not be as supportive of or knowledgeable about the GRADS program as prior administrators, the state office prioritizes early and frequent engagement with the new administrators similar to their initial engagement efforts for new sites. They also provide school leaders at each site with an annual fact sheet on GRADS students’ outcomes at their site. PED and the state GRADS office invite superintendents and principals to attend the biannual in-person trainings and give them a platform to share their experiences through roundtable and panel discussions.

### Lesson Four: Programs Should Collect Consistent, Longitudinal Data Across Sites to Measure Program Outcomes Over Time

During the PAF funding period, GRADS program staff collected some data on the participants at each site and reported this information to PED and the state GRADS office. Using a paper form, site staff tracked the number of participants in the program each year and collected data on their repeat pregnancies and senior graduation rates, based primarily on staff knowledge of these outcomes (although students were also able to report on their number of children at the beginning and end of the program). State staff used this data to calculate repeat pregnancy rates and senior graduation rates for each site and across sites, monitor the program, and make program improvements.

Although this data can provide some insights into how the program may influence participants’ outcomes, it does not come from a rigorous evaluation. In particular, state staff did not have a comparison group to serve as a counterfactual for these outcomes, and site staff did not track outcomes for participants longitudinally or if they dropped out of the program. Programs should consider how to collect reliable longitudinal data, including on a comparison group of youth similar to their own participants. Such data allows for more rigorous assessment of program impacts. As described in Zief et al. in this issue, another PAF grantee used administrative data across multiple agencies to track outcomes for participants and comparison group students, which was a low-cost evaluation option and worked well because the grantee could access strong existing administrative data systems. Alternatively, programs can collect survey or interview data from participants and comparison group youth, which may be more time- or labor-intensive for programs but allow for the collection of tailored information about program outcomes, including those tied specifically to program logic models.

## Conclusion

The experiences of implementing the GRADS program can help inform the development, implementation, and evaluation of multifaceted programs that serve EPY across many sites. Most EPY face challenges with finishing high school and require targeted support to address those challenges. The GRADS program is structured to support these common challenges through case management; child care services or referrals; linkages to health care; support for young fathers; and a classroom-based intervention that covers topics related to family planning, parenting, and college and career readiness. During the PAF funding expansion period, the GRADS program was implemented at 36 sites across New Mexico, an effort that required strong centralized support from a statewide GRADS office, along with flexibility to address the specific needs of each site. Other practitioners or policymakers interested in implementing a statewide, multifaceted, and school-based program for EPY should consider lessons learned from the GRADS program: (1) allow for variation to the program model across sites to provide meaningful services for EPY at each site because each site will have unique needs based on its specific population of EPY; (2) have clear lines of communication and provide centralized monitoring and support to site staff; (3) invest in building and maintaining principal and superintendent support for the program to strengthen program sustainability over time; and (4) collect data longitudinally and aim to assess comparable control participants to allow for rigorous assessment of whether the program is working as intended.

Although this paper describes a potentially promising school-based support program for EPY, it has limitations. First, the program has not been evaluated in a rigorous impact or implementation study. The findings from these lessons are based on the experiences of program administrators; youth or site-level staff may have different lessons learned. Finally, the article describes one program, implemented in one state, and it is not clear if the program components would be well suited to different state contexts.

Despite these limitations, this article provides important information about the components of a school-based program for EPY. More research is needed to evaluate the New Mexico GRADS program and other school-based programs. Future research should include an experimental design to assess multiple potential outcomes associated with the program.

## Footnotes

1 During the PAF funding period, three sites did not offer the classroom intervention but had onsite case management to assess students’ needs and link to services, including onsite child care and SBHCs.

2 In one of these sites, the SBHC was at a different, nearby school campus with which the site had a close relationship.

3 Six sites, including one of the sites that began GRADS in the PAF funding period, stopped implementing GRADS during the PAF funding period.

4 This research was conducted in accord with prevailing ethical principles. However, this research did not receive Institutional Review Board approval because it includes the use of program enrollment forms and information provided by program administrators, including the article authors. Thus, releasing information would not be harmful to the subjects.
